# Differential Requirements in Endocytic Trafficking for Penetration of Dengue Virus

**DOI:** 10.1371/journal.pone.0044835

**Published:** 2012-09-07

**Authors:** Eliana G. Acosta, Viviana Castilla, Elsa B. Damonte

**Affiliations:** Laboratorio de Virología, Departamento de Química Biológica, Facultad de Ciencias Exactas y Naturales, Universidad de Buenos Aires, Buenos Aires, Argentina; CNRS, France

## Abstract

The entry of DENV into the host cell appears to be a very complex process which has been started to be studied in detail. In this report, the route of functional intracellular trafficking after endocytic uptake of dengue virus serotype 1 (DENV-1) strain HW, DENV-2 strain NGC and DENV-2 strain 16681 into Vero cells was studied by using a susceptibility to ammonium chloride assay, dominant negative mutants of several members of the family of cellular Rab GTPases that participate in regulation of transport through endosome vesicles and immunofluorescence colocalization. Together, the results presented demonstrate that in spite of the different internalization route among viral serotypes in Vero cells and regardless of the viral strain, DENV particles are first transported to early endosomes in a Rab5-dependent manner. Then a Rab7-dependent pathway guides DENV-2 16681 to late endosomes, whereas a yet unknown sorting event controls the transport of DENV-2 NGC, and most probably DENV-1 HW, to the perinuclear recycling compartments where fusion membrane would take place releasing nucleocapsid into the cytoplasm. Besides the demonstration of a different intracellular trafficking for two DENV-2 strains that shared the initial clathrin-independent internalization route, these studies proved for the first time the involvement of the slow recycling pathway for DENV-2 productive infection.

## Introduction

Dengue virus (DENV) is a member of the family *Flaviviridae* transmitted to humans by mosquitoes of the genus *Aedes*. The virion is an enveloped particle containing single-stranded RNA positive genome and three structural proteins (envelope E, membrane M and capsid C proteins). There are four antigenically related but distinct serotypes (DENV-1 to DENV-4) which co-circulate in tropical and subtropical regions. As a consequence of the increased re-emergence and rapid spread of flavivirus infections in the last decades, dengue is currently endemic in more than 100 countries and is considered the most prevalent arthropod-borne disease worldwide [1], [2]. Given the lack of vaccines and specific therapies against DENV infection, there is an urgent requirement to find and characterize new potential targets for antiviral chemotherapy.

Virus entry is an attractive target for antiviral intervention that gained attention in recent years to block the initiation of infection [3], [4]. As other flaviviruses, DENV enters mammalian and mosquito cells by receptor-mediated endocytosis [5]–[10]. The intravesicular low pH of endosomes triggers a conformational change of E glycoprotein leading to fusion between viral envelope and endosome membrane and release of nucleocapsid into the cytoplasm [11], [12]. In contrast to the certain requirement of acid pH for E-mediated fusion in entry, the precise intracellular pathway for DENV internalization and penetration until fusion and uncoating is at present not fully understood and information is controversial. Recent evidence suggests that the serotype DENV-2 is internalized in HeLa [5], C6/36 [7]–[8], BSC-1 [13] and Huh7 [14] cells in the clathrin-dependent endocytic pathway. But, in Vero cells a differential route of entry was demonstrated according to virus serotype: DENV-1 enters also by clathrin-mediated endocytosis whereas DENV-2 is internalized through a non classical clathrin- and caveolin- independent process [9]. This variable behavior among DENV serotypes for entry into Vero cells was of particular interest considering that Vero cells are widely used to make vaccines, including flavivirus vaccines, and also represent the usual system to test antiviral candidates against DENV. Furthermore, differences were also reported in the intracellular location of viral fusion for different strains of DENV-2: Krishnan et al. [5] reported that DENV-2 strain NGC virions fused predominantly from early endosomes in HeLa cells, but other studies demonstrated fusion from late endosomes for DENV-2 strains 16681 and PR159 S1 in C6/36 HT and BSC-1 cells, respectively [8], [13].

Given the differential clathrin-dependence shown for the infectious entry of DENV-1 and DENV-2 in Vero cells as well as the reported differences in the subcellular location for distinct DENV-2 strains fusion, in the present study we analyzed the route of functional intracellular trafficking for DENV-1 and two different strains of DENV-2 after endocytic uptake in order to establish and compare under the same experimental conditions their requirements for productive fusion and its relationship with the initial internalization pathway.

## Materials and Methods

### Cells and Viruses

The African green monkey kidney cell line Vero (ATCC CCL-81) was grown at 37°C in Eagle’s minimum essential medium (MEM) (GIBCO BRL, USA) supplemented with 5% calf serum (GIBCO BRL, USA) and 50 µg/ml gentamycin. The C6/36 mosquito cell line from *Aedes albopictus* adapted to grow at 33°C was cultured in L-15 medium (Leibovitz) (GIBCO BRL, USA) supplemented with 0.3% tryptose phosphate broth, 0.02% glutamine, 1% MEM non-essential amino acids solution, 5% fetal calf serum (GIBCO BRL, USA) and 50 µg/ml gentamycin. For maintenance medium (MM) of L-15 and MEM serum concentration was reduced to 1.5%.

DENV-2 strain New Guinea C (NGC) and the clinical isolates of DENV-1 ARG9920 and ARG0044 were kindly provided by Dr. A. Mitschenko, Hospital R. Gutiérrez, Buenos Aires, Argentina; DENV-1 strain Hawaii (HW) and the DENV-2 clinical isolates 67655 and 67702 were obtained from Dr. D. Enría, Instituto Nacional de Enfermedades Virales Humanas, Pergamino, Argentina; DENV-2 strain 16681 was kindly provided by Dr. A. Gamarnik, Fundación Instituto Leloir, Buenos Aires, Argentina. All DENV virus stocks were prepared in C6/36 cells and titrated by plaque forming units (PFU) in Vero cells. Junín virus (JUNV) strain IV4454 was propagated and titrated by PFU in Vero cells.

### Antibodies and Reagents

The mouse monoclonal antibody reactive against the E glycoprotein of the four DENV serotypes was purchased from Abcam (Cambridge, UK). The mouse monoclonal antibody specific for DENV-2 C protein (clone 6F3.1) [15] and the mouse monoclonal antibody SA02-BG12 reactive against the nucleoprotein NP of JUNV [16] were kindly provided by Dr. J. Aaskov (Univesity of Queensland, Australia) and Dr. A. Sanchez (Center for Disease Control, Atlanta, USA), respectively. The rabbit polyclonal anti-Rab5 antibody was purchased from Cell Signaling (USA). Goat anti-mouse IgG conjugated to fluorescein isothiocyanate (FITC) or rhodamine (TRITC) were purchased from Sigma-Aldrich (USA). TRITC-human transferrin was from Molecular Probes (USA). Ammonium chloride, chlorpromazine, acridine orange and wortmannin were purchased from Sigma- Aldrich (USA).

### Inhibition of DENV Multiplication by Pharmacological Inhibitor Treatment

The effect of chlorpromazine on DENV-1 and DENV-2 was determined by a virus-yield inhibition assay as previously described [9]. Briefly, monolayers of Vero cells were treated for 2 h with chlorpromazine 50 µM and then infected at a multiplicity of infection (MOI) of 0.1 in the presence or absence of the compound. Virus inocula were removed after 1 h of infection at 37°C, and then cultures were washed with PBS and further incubated at 37°C in MM without compound. Extracellular virus yields were determined at 48 h post-infection (p.i.) by plaque assay.

### Fusion Kinetics by Ammonium Chloride Treatment and Visualization of E and C Protein Subcellular Distribution

Vero cells (5×10^5^) were infected with 100–200 PFU/well of DENV-1, DENV-2 or JUNV. After 1 h adsorption at 4°C, virus inoculum was removed, cell monolayers were washed twice with ice cold PBS and incubated with MM prewarmed at 37°C to initiate virus internalization. Ammonium chloride (50 mM final concentration) was added at the indicated times after addition of warmed medium and kept throughout the infection. After 3 h of incubation at 37°C, cells were washed with PBS and treated with citrate buffer (40 mM citric acid, 10 mM KCl, 135 mM NaCl pH 3) for 1 min to inactivate adsorbed but not internalized virus. Then, cells were washed with PBS and covered with MM containing 1% methylcellulose. Plaques were counted at 6 days p.i.

To assess the effect of ammonium chloride on intracellular vesicle pH acidification, Vero cells treated or not with the compound during 1h at 37°C were stained with 1 µg/ml acridine orange in MM without serum for 15 min at 37°C. Cells were washed twice with PBS and visualized under a fluorescence microscope.

For visualization of E and C protein subcellular distribution by immunofluorescence, Vero cells were adsorbed with DENV-2 for 1 h at 4°C at an MOI of 10. After removal of virus inoculum, cells were washed with ice cold PBS and covered with prewarmed medium to initiate internalization. Cultures were incubated at 37°C for the indicated time periods and fixed with methanol for 10 min at −20°C. Then, cells were washed with PBS and stained for DENV internalization with a monoclonal antibody against E or C proteins followed by FITC-labeled goat anti-mouse IgG. After a final washing with PBS, cells were mounted in a glycerol solution containing 1,4-diazabicyclo [2,2,2]octane (DABCO) and visualized under a fluorescence microscope (Olympus BX51) with a 100× objective lens.

### Transfections

The green fluorescent protein (GFP) tagged constructs of Rab 5 and Rab7 wild-type and dominant negative (DN) mutants S35N and T22N, respectively, were kindly provided by Dra. M. I. Colombo (Universidad Nacional de Cuyo, Argentina). The GFP tagged constructs of human wild-type and Q64L mutant of Rab22 were a generous gift of Dr. P. D. Stahl (Washington University School of Medicine, USA), and the GFP tagged constructs of Eps15 GFP-EH29 (DN mutant) and GFP-DIIIΔ2 (control) were kindly provided by Dra. C. Shayo (IBYME, Argentina). The Green-lantern tagged constructs of Rab11 wild-type and S25N DN mutant were provided by Dr. G. Whittaker (Cornell University, USA).

Vero cells grown on cover slips until 70% confluency, were transfected with each construct using Lipofectamine 2000 reagent (Invitrogen, USA) as previously described [9]. Briefly, 4 µg of each construct was diluted in 50 µl Opti-MEM (GIBCO BRL, USA) and combined with 50 µl Opti-MEM containing 2.5 µl lipofectamine. After 40 min of incubation at room temperature, the DNA-liposome complexes were added to the cells and cultures were incubated for 6 h at 37°C. At this time, medium was replaced by MM and cells were incubated till 24 h post-transfection. Transfection efficiency was dependent on the construct: 50% efficiency of transfection was achieved with Rab 5 and Rab 11 plasmids, while 30% and 15% efficiency was obtained with Rab 22 and Rab 7 constructs, respectively.

### Immunofluorescence and Colocalization Assays in Transfected Cultures

For visualization of internalized viral particles, 24 h-transfected cell cultures were infected with DENV-1 or DENV-2 at an MOI of 10 PFU/cell and after 1 h of infection cells were fixed with methanol for 10 min at −20°C. After methanol fixation, cells were washed with PBS, stained for DENV internalization with a monoclonal antibody against the E glycoprotein followed by TRITC-labeled goat anti-mouse IgG and processed for fluorescence visualization with a 100× objective lens.

For visualization of viral antigen production 24 h-transfected cell cultures were infected with DENV-1 or DENV-2 at an MOI of 1 PFU/cell and after 24 h infection cultures were fixed and stained for DENV multiplication with a monoclonal antibody against the E glycoprotein followed by TRITC-labeled goat anti-mouse IgG as above. The percentage of infection of transgene-expressing cells was calculated by scoring the number of cells positive for viral antigen from approximately 250 transfected cells with comparable levels of transgene expression.

For colocalization studies 24 h transfected cells were infected with DENV-2 (MOI = 10) and incubated at 4°C for 60 min. Then cells were washed with cold PBS, covered with pre-warmed media and shifted to 37°C for 15 min. Fixation and immunofluorescence assay for C protein staining was performed as above. Cells were visualized using a confocal microscope (Olympus Fluoview) with a 60× objective lens.

### Immunofluorescence and Colocalization Assays in Wortmannin Treated Cultures

Vero cells were pretreated 1 h with wortmannin 100 nM and infected with DENV-2 NGC or 16681 in the presence or absence of the drug. After 10 or 60 min of incubation at 37°C cells were fixed with methanol. Rab5 and C proteins were revealed by indirect immunofluorescence using FITC-conjugated and TRITC-conjugated secondary antibodies, respectively. Cells were visualized using a confocal microscope (Olympus Fluoview) with a 60× objective lens and the Mander’s overlap coefficient was calculated, from 20 cells using the image J software, in order to estimate the degree of colocalization.

## Results

### Entry and Fusion Kinetics of DENV-1 and DENV-2

**Figure 1:**
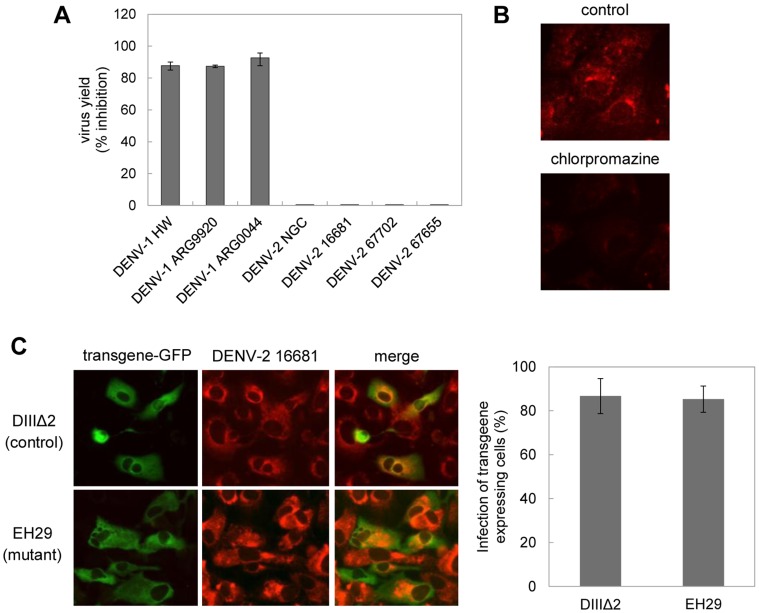
Effect of blockade of clathrin-mediated endocytosis on DENV-1 and DENV-2 strains. A. Cells were treated with 50 µM chlorpromazine and then infected with reference strains and clinical isolates of DENV-1 or DENV-2. Virus yields were quantified by PFU at 48 h p.i. and results are expressed as % of inhibition of virus multiplication with respect to a control of infected cells without drug treatment. Each value is the mean±SD of three independent experiments. B. Cells were treated with 50 µM chlorpromazine or left untreated and incubated with TRITC-labelled transferrin. C. Cells transiently transfected with the constructs GFP-DIIIΔ2 or GFP-EH29 were infected with DENV-2 strain 16681. After 24 h cells were fixed and viral antigen expression was visualized by immunofluorescence staining using mouse anti-E glycoprotein antibody and TRITC-labelled anti-mouse IgG.

By using biochemical and molecular inhibitors we have shown previously that the endocytic entry of DENV-1 strain HW in Vero cells is clathrin-mediated whereas DENV-2 strain NGC enters through a pathway independent of clathrin and caveolin [9]. We corroborated that the differential properties between DENV-1 and DENV-2 for endocytic pathway of entry into Vero cells was not privative of these two strains but it was preserved in other reference strains and recent Argentinian clinical isolates of both serotypes: DENV-1 infection was inhibited in the presence of chlorpromazine, a pharmacological inhibitor of clathrin-mediated endocytosis, whereas no effect of this compound was observed against DENV-2 infection, independently of the strain (Fig. 1A). The internalization of TRITC-labelled transferrin, a typical ligand known to enter into the cell by clathrin-mediated endocytosis, was used as a control assay to assess the chlorpromazine action was exerted on this endocytic route. In control cells, a bright cytoplasmic fluorescence was observed whereas cells treated with chlorpromazine showed a very weak fluorescence only at cell surface indicating the blockade of transferring uptake (Fig. 1B).

The lack of participation of the clathrin pathway in the infective entry of DENV-2 was also assessed by the overexpression of a dominant negative mutant of the clathrin coat-associated protein Eps15, which specifically interferes with clathrin-coated pit assembly at the plasma membrane without affecting clathrin-independent endocytic pathways [17]. As shown in Fig. 1C for DENV-2 16681, the presence of the mutant protein did not significantly affect infection since similar signals for merge images were seen in dominant and control transfected cells (Fig. 1C). The percentage of infection in transgene-expressing cells, determined by scoring cells expressing viral antigens, was similar in cultures transfected with the mutant or control protein (85.3% and 86.7% of DENV-2 positive cells, respectively).

**Figure 2:**
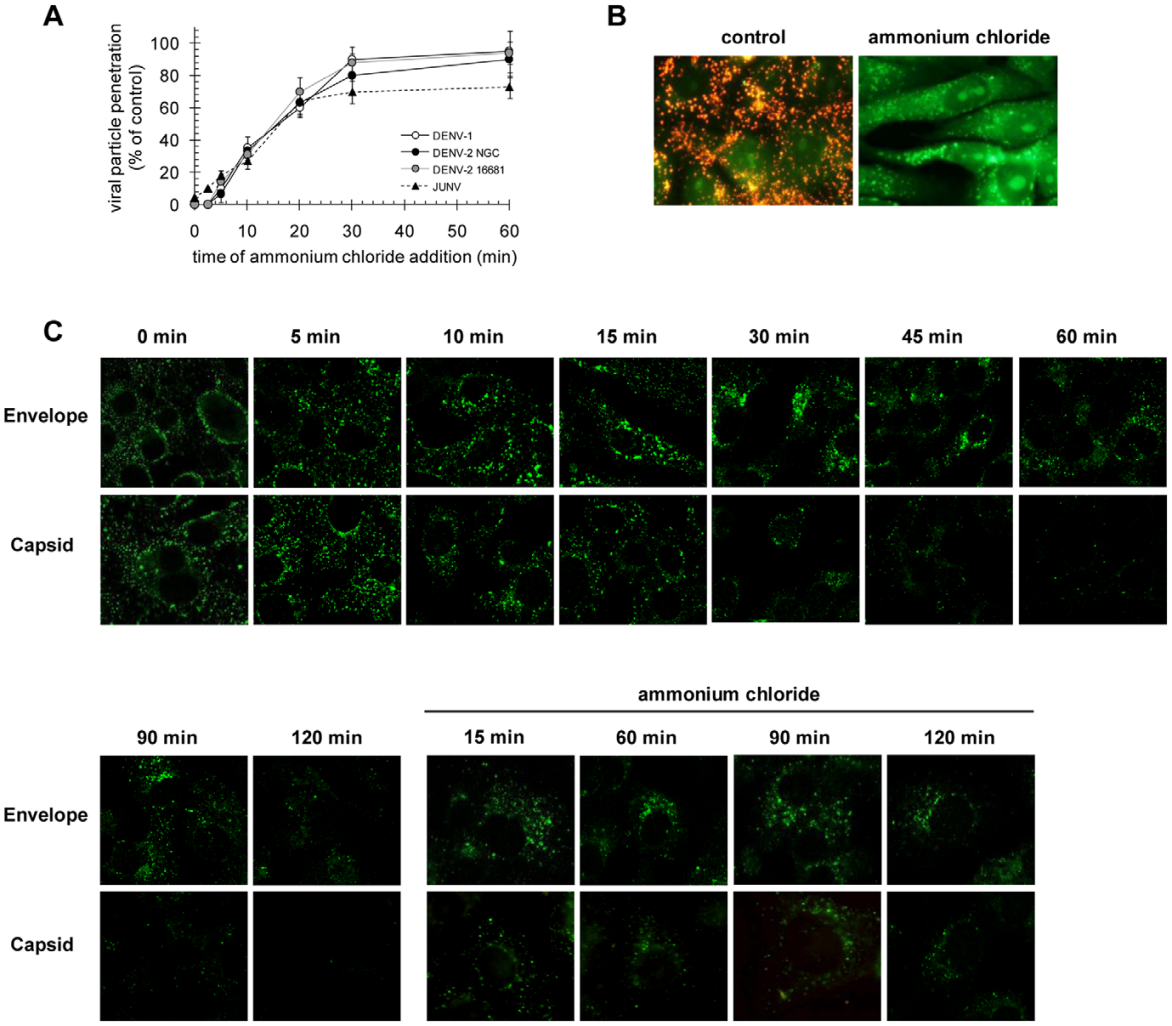
Time course of DENV penetration. A. 100–200 PFU/well of DENV-1 HW, DENV-2 NGC, DENV-2 16681 or JUNV were bound to cells at 4°C and then allowed to internalize after rapid warming to 37°C. Ammonium chloride was added at different time points to inhibit penetration and infection. Cells were further incubated at 37°C for 3 h, then extracellular virus was inactivated and cultures were overlaid with plaquing medium. The infection levels observed were normalized to the level in control cells without ammonium chloride. Each point is the mean±SD of three independent experiments. B. Cells were treated or not with 50 mM ammonium chloride and then stained with acridine orange. C. DENV-2 NGC was adsorbed to Vero cells during 1 h at 4°C in the presence or absence of 50 mM ammonium chloride and then cultures were shifted to 37°C. At different time intervals cultures were fixed and processed to reveal C and E protein by immunofluorescence using mouse anti-C and mouse anti-E antibodies followed of FITC-labelled anti-mouse IgG, respectively.

Hence, the reference strains HW of DENV-1 and NGC and 16681 of DENV-2 were further studied to characterize the intracellular trafficking of DENV in Vero cells from the initial uptake until the time that membrane fusion is triggered by acid endosomal pH. First, we determined the kinetics of viral penetration using a protocol to determine the time required for DENV to become resistant to the lysosomotropic agent ammonium chloride [18]–[20]. The treatment of cells with this drug raises the endosomal pH instantaneously and prevents low pH-dependent processes without cell toxicity. Virus was bound to cells at 4°C, unbound virus was thoroughly washed, and then cells were warmed to 37°C and incubated for different time intervals before addition of medium containing ammonium chloride to block further penetration. After 3 h of incubation with this compound, non-internalized virus was totally eliminated by a brief treatment of cells with citrate buffer and monolayers were overlaid with plaquing medium. As a benchmark, we determined the penetration kinetics of the arenavirus JUNV, a virus that penetrates in Vero cells at pH 5.5 in late endosomes [21], [22]. As seen in Fig. 2A, the three DENV viruses exhibited the same kinetics profiles of susceptibility to ammonium chloride. Penetration started at 5 min post-binding and more than 80–90% of virions reached the low pH-dependent activation during the period of 30 min post-warming. For the three strains, the half time for ammonium chloride resistance, equivalent to viral escape from endosomes, was in the range 14–16 min. This half time of membrane penetration resembles that of the late penetrating arenavirus JUNV (Fig. 2A), suggesting a membrane fusion in late endosomes for DENV [18], [19], [23], and it is in accordance with the mean fusion time in late endosomes reported for DENV-2 strain PR159 S1 [6], [13]. To ensure that the drug treatment effectively inhibits acidification of endosomal vesicles in our cell system, acridine orange staining was performed. Untreated control cells showed cytoplasmic orange fluorescence of acid compartments, but cells treated with ammonium chloride did not show this pattern (Fig. 2B).

The kinetics of virus internalization into Vero cells was also analysed by monitoring the distribution of capsid and envelope proteins in infected cells within the initial period of incubation at 37°C after virion binding at 4°C. It has been described for other enveloped viruses such as vesicular stomatitis virus and Semliki Forest virus that when the virus envelope-endosomal membrane fusion takes place the genome is released to the cytoplasm together with the capsid protein and the envelope proteins remain associated to the endosome for further degradation in the endolysosomes [24], [25]. At 5 min post-adsorption, a bright dotted fluorescence pattern for both C and E proteins was observed in the cytoplasm (Fig. 2C). In agreement with the kinetics of fusion determined by infectivity resistance to ammonium chloride (Fig. 2A), the amount of fluorescence corresponding to C protein was highly reduced at 10–15 min, concentrated in the perinuclear zone, and resulting almost undetectable from 30 min onwards. By contrast, the dotted fluorescence of E glycoprotein remained between 5 and 45 min post-attachment, with an increasing perinuclear distribution and starting to disappear after 90 min (Fig. 2C). This differential pattern change between C and E proteins suggests that once fusion starts C is liberated to the cytoplasm, process completed after 30 min, while E remains inserted in the endosome membrane until degradation is fulfilled, probably in endolysosomes, at 120 min after infection. This conclusion was reinforced by repeating the assay but in the presence of ammonium chloride. In the absence of fusion both C and E proteins behave in a similar way showing an accumulation mostly in the perinuclear area even after 120 min of internalization (Fig. 2C).

### Transit of DENV through Early and Late Endosomes

To further investigate the cellular compartments involved in intracellular fusion for DENV penetration, we then examined the roles of the small Rab5 and Rab7 GTPases, known to be key regulators in vesicular trafficking to early and late endosomes, respectively [26], [27]. To this end, the DN mutants of Rab5 S34N and Rab7 T22N, which have been validated in entry studies with different enveloped viruses [5], [19], [28], were employed.

**Figure 3:**
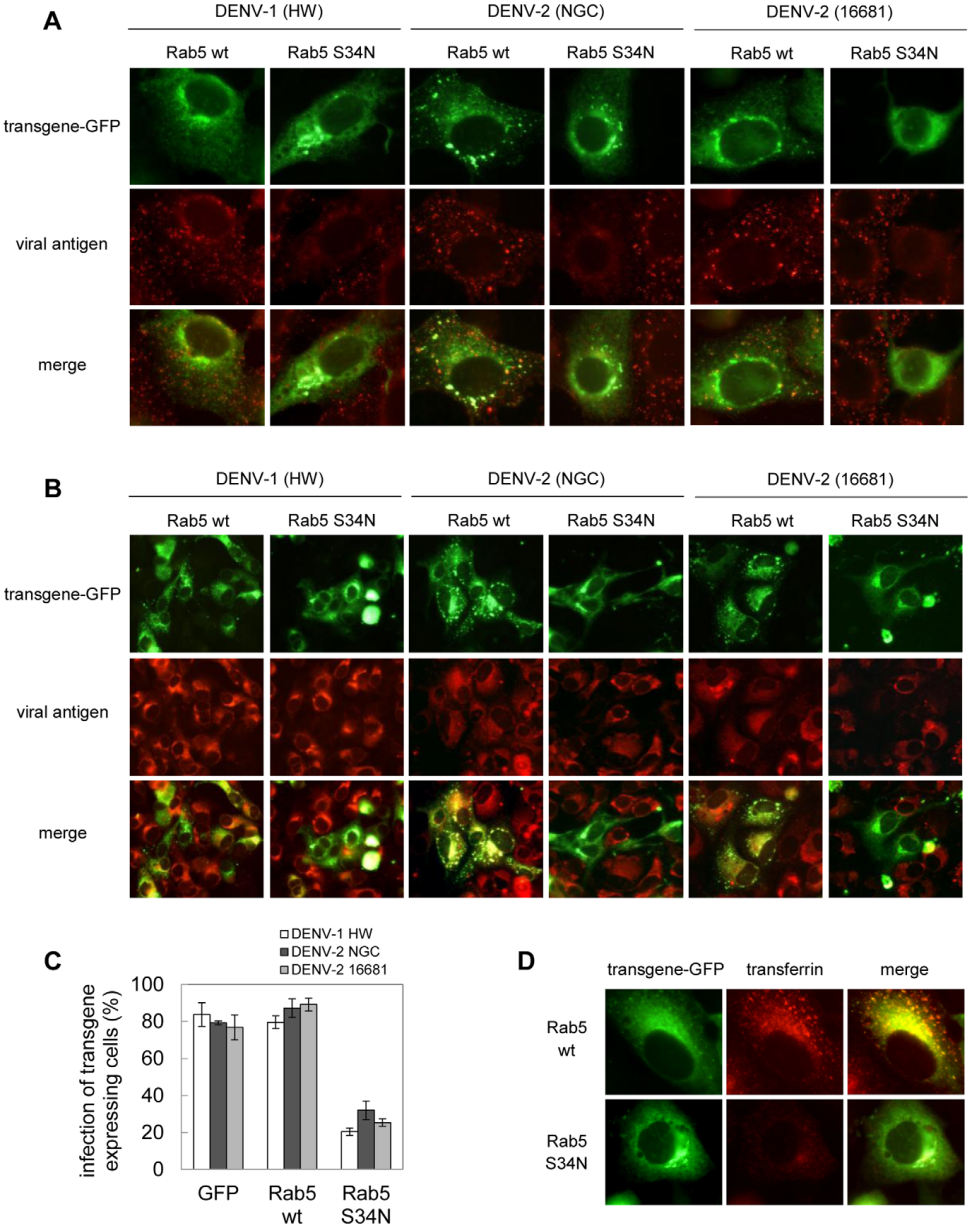
Transport of DENV particles to early endosomes. A. Cells transiently transfected with the GFP-tagged versions of Rab5 wt and S34N, and the plasmid pGFP-C1 were infected with DENV-1 HW, DENV-2 NGC or DENV-2 16681 (MOI:10 PFU/cell). After 1 h of infection, cells were fixed and processed to visualize GFP transgene expression and internalized viral particles by immunofluorescence staining using mouse anti-E glycoprotein antibody and TRITC-labelled anti-mouse IgG. B. Cells transfected as in A) were infected with DENV-1 HW, DENV-2 NGC or 16681 (MOI:1 PFU/cell). After 24 h of infection cultures were fixed and immunofluorescence staining was performed as in A. C. For quantification of samples shown in B, 250 transfected cells with similar levels of GFP expression were screened and cells positive for viral antigen were scored. D. Cells transfected as in A were then incubated with TRITC-labelled transferrin during 30 min. Then, cells were fixed and fluorescence was visualized.

First, cells were transfected with the control vector pGFP-C1 or constructs expressing GFP-tagged versions of wt and DN form of Rab5 to analyze the transit to early endosomes. After 24 h of transfection cells were infected with DENV-1 or DENV-2 for 1 h at 37°C and then processed to detect GFP expression and internalized viral antigen by indirect immunofluorescence staining of E glycoprotein. When Vero cells were transfected with the wt plasmid, both DENV-1 and DENV-2 virions were internalized in endocytic vesicles exhibiting a speckled and strong virus antigen staining within the cytoplasm and superposition of GFP autoflorescence and viral protein immunofluorescence (Fig. 3A). In contrast, the expression of the DN affected virus internalization, evidenced by a slight and disperse red fluorescence. To examine the effect of blockade in Rab5-mediated transport on productive infection, transfected cells were allowed to be infected during 24 h before proceeding to cell fixation and staining. The overexpression of Rab5 DN S34N reduced the infection by DENV-1 and the two strains of DENV-2 approximately to 70%, indicating the requirement of the functionality of Rab5 and, consequently, transport of viruses to early endosomes for successful infection (Fig. 3 B, C). The functionality of Rab5 transgenes in Vero cells was ensured using TRITC-labelled transferrin as control [29]. As expected the overexpression of Rab5 DN S34N reduced the accumulation of transferrin in comparison with cells expressing Rab5 wt, with minor fluorescence intensity in cytoplasm and failure in the superposition of both proteins (Fig. 3 D).

**Figure 4:**
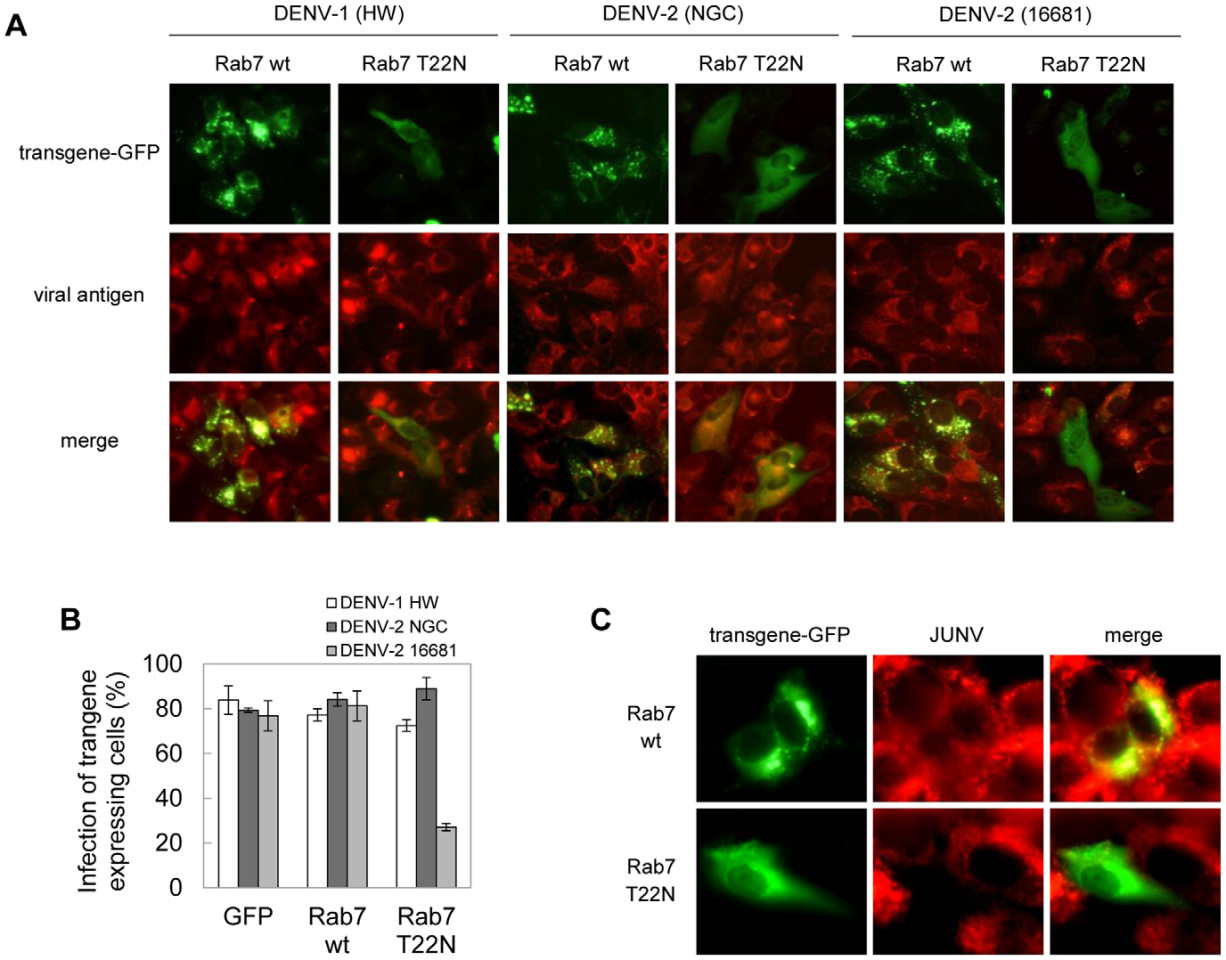
Transport of DENV particles to late endosomes: Rab7 dependence. A. Cells transiently transfected with the GFP-tagged versions of Rab7 wt and DN T22N and the plasmid pGFP-C1 were infected with DENV-1 HW, DENV-2 NGC or 16681. After 24 h of infection cultures were fixed and processed to visualize GFP transgene expression and viral antigen by immunofluorescence staining using mouse anti-E glycoprotein antibody and TRITC-labelled anti-mouse IgG. B. For quantification of samples shown in A, 250 transfected cells with similar levels of GFP expression were screened and cells positive for viral antigen were scored. C. Cells transiently transfected as in A were infected with JUNV. At 24 h p.i. cells were fixed and infection was assessed by immunofluorescence using mouse anti-JUNV NP antibody and TRITC-labelled anti-mouse IgG.

Next, the requirement of transport to late endosomes was studied by transfection of Vero cells with GFP-tagged versions of wt and DN mutans of Rab7. The expression of Rab7 DN T22N showed a differential inhibitory effect against DENV strains: it did not affect the infection of Vero cells with DENV-1 HW or DENV-2 NGC, but a 65% reduction on viral antigen expression in cells infected with DENV-2 16681 was observed (Fig. 4A, B). The functionality of Rab7 transgenes in Vero cells was ensured using JUNV, an arenavirus reported to traffick through Rab7 dependent late endosomes for entry into the cell [22] as control. As shown in Fig. 4C, JUNV nucleoprotein expression was diminished in the presence of Rab7 DN T22N while no inhibition of JUNV protein was observed with Rab7 wt. These results demonstrate that the traffic of DENV particles from early to late endosomes should be required for DENV-2 16681 strain whereas the efficient infection with DENV-1 HW and DENV-2 NGC occurs when Rab7-mediated transport is blocked.

**Figure 5:**
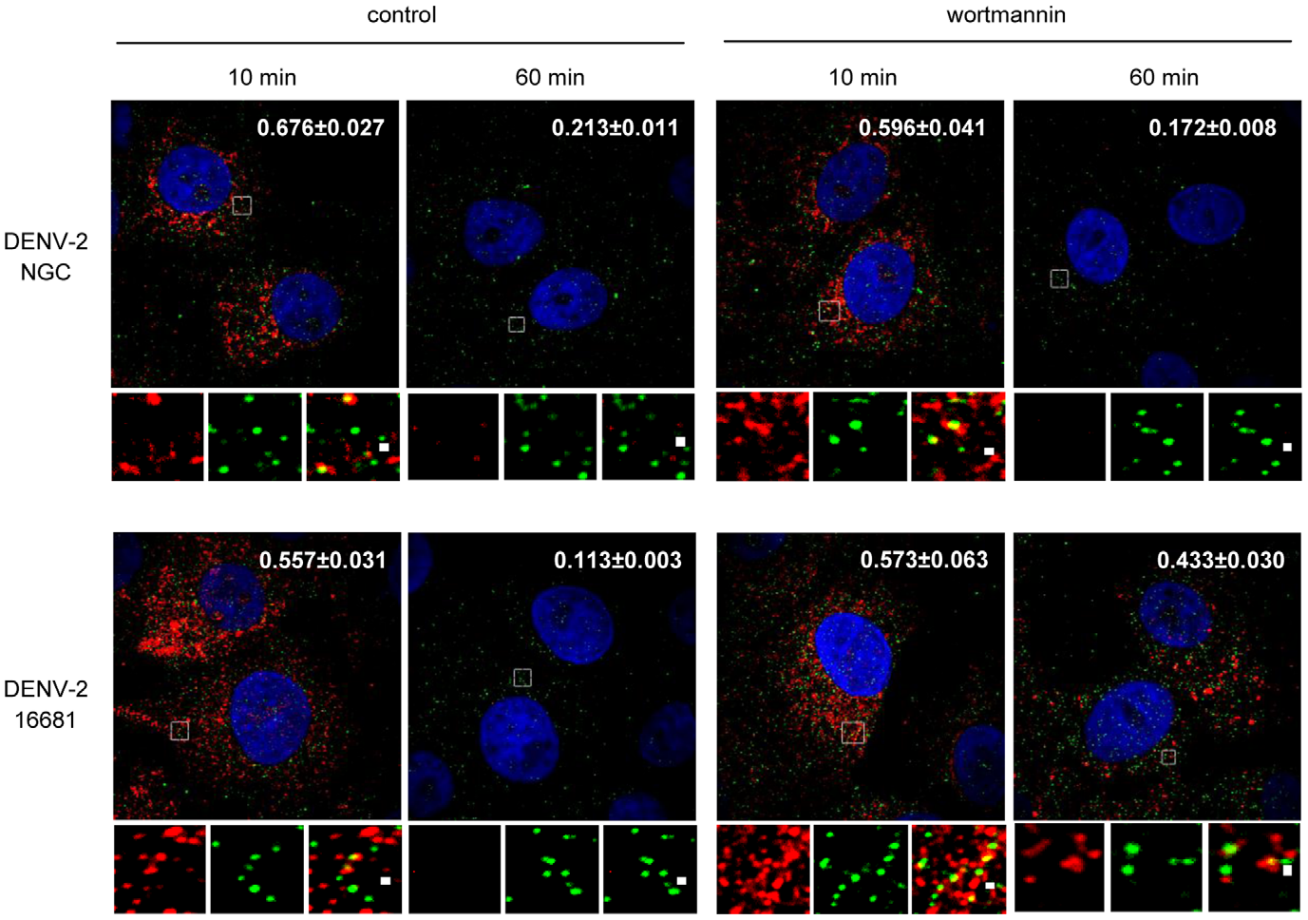
Transport of DENV particles to late endosomes: effect of wortmannin. Vero cells were infected with DENV-2 NGC or 16681 in the presence of wortmannin 100 nM and after 10 or 60 min at 37°C cultures were fixed. Rab5 was revealed using a rabbit polyclonal antibody followed by FITC-conjugated secondary antibodies, while C protein was revealed using a mouse monoclonal antibody followed by incubation with TRITC-conjugated secondary antibodies. Enlarged details of single and merged channels of the boxed areas are shown. The degree of colocalization was estimated by calculating the Mander’s overlap coefficient from 20 cells using the application imageJ. Values are indicated in each picture.

Another experimental approach was utilized to assess the differential requirement for transport to late endosomes between the two strains of DENV-2. Wortmannin is a potent inhibitor of phosphatidylinositol-3-kinases (PI3K) class I and III which has been shown to block the maturation from early to late endosomes interfering further transport between them [30], [31]. In particular for viruses, it was described the accumulation of human rhinovirus type 2 and adenovirus type 2 escape defective temperature-sensitive mutants in early endosomes after cell treatment with wortmannin [32], [33]. Then, we evaluated the effect of this inhibitor on the entry of DENV-2 NGC and DENV-2 16681: Vero cells were treated with wortmannin and then infected with both strains at high MOI. The colocalization of the capsid protein at 10 and 60 min p.i. with the early endosomal marker Rab5 was followed by immunofluorescence. In accordance with kinetics assay shown in Fig. 2A, after 10 min of internalization the C protein of both viruses was detected in the endosomal compartments in control and treated cells showing a dotted fluorescence that colocalize with Rab5 (Fig. 5). At 60 min, the C protein fluorescence disappeared in control cells infected with both DENV-2 strains due to virus uncoating. The presence of wortmannin did not affect DENV-2 NGC penetration, as shown by the loss of capsid protein immunofluorescence after 60 min of infection similarly to control cells, but in cells infected with DENV-2 16681 the dotted fluorescence pattern of C protein still remained colocalizing with Rab5, confirming that the blockade in early endosome maturation prevented the cellular trafficking of this virus strain and its fusion.

### Transit of DENV through Recycling Endosomes

**Figure 6:**
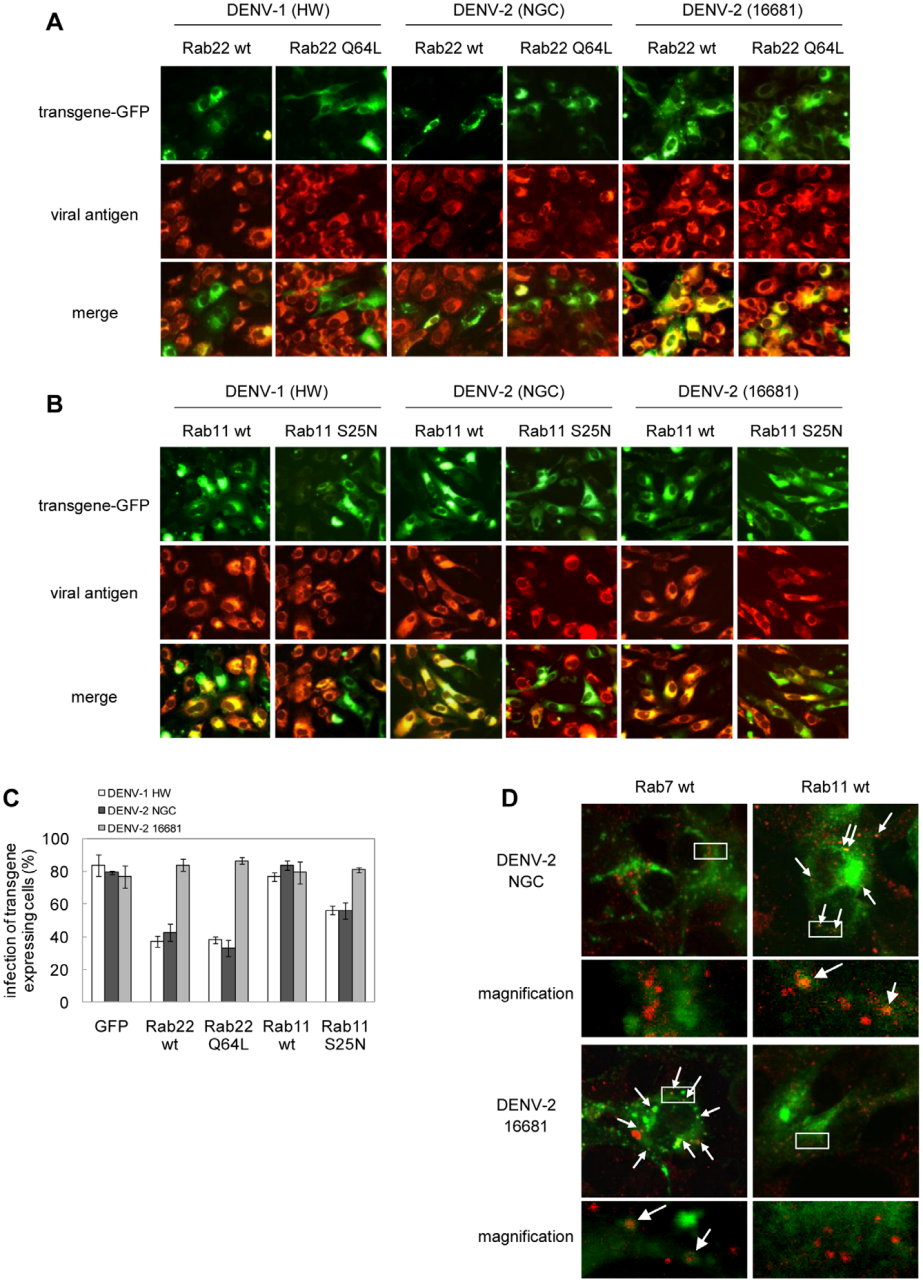
Transport of DENV particles to recycling endosomes. Cells transiently transfected with the GFP-tagged versions of Rab22 wt and Q64L (A), Rab11 wt and S25N (B) and the plasmid pGFP-C1 were infected with DENV-1 HW, DENV-2 NGC or 16681. After 24 h of infection cultures were fixed and processed to visualize GFP transgene expression and viral antigen by immunofluorescence staining using mouse anti-E glycoprotein antibody and TRITC-labelled anti-mouse IgG. C. For quantification of samples shown in A and B, 250 transfected cells with similar levels of GFP expression were screened and cells positive for viral antigen were scored. D. Cells expressing GFP-Rab7 wt or Green Lantern-Rab11 wt were infected at 24 h post-transfection with DENV-2 NGC or 16681 during 60 min at 4°C and then shifted at 37°C. After 15 min cells were fixed and processed to visualize GFP transgene expression and viral antigen by immunofluorescence staining using mouse anti-C glycoprotein antibody and TRITC-labelled anti-mouse IgG. Cells were visualized with a confocal microscope.

The failure of Rab7 DN T22N to inhibit DENV-1 HW and DENV-2 NGC infection indicates that these viruses are not transported to late endosomes. However, the kinetics profile of viral fusion (Fig. 2A) is not consistent with a fusion event between viral and cellular membranes within early endosomes. After entering the early endosomes, also called sorting endosomes due to their functional role, there are three known possible destinations for internalized molecules: late endosomes, rapid recycling to the plasma membrane or slow recycling to the perinuclear recycling endosomes [34], [35]. Thus, the possible participation of recycling endosomes as the endocytic vesicles for fusion and release of nucleocapsids to the cytoplasm was next analyzed. To this end, we evaluated the involvement of Rab22 in DENV-1 and DENV-2 infection of Vero cells. It has been reported that the overexpression of both either Rab22 wt or the mutant version Rab22 Q64L causes a dramatic enlargement of early endosomes and inhibits the transport of transferrin to recycling endosomes, whereas the overexpression of these Rab GTPases does not affect the internalization or transport of ligands following the degradative pathway through late endosomes/lysosomes [36]. Vero cells were transfected with plasmids expressing the human Rab22 wt and the mutant Rab22 Q64L, both tagged with GFP, together with a control culture transfected with the vector pGFP-C1, and 24 h after transfection cells were infected with DENV-1 HW, DENV-2 NGC or DENV-2 16681. The expression of both, Rab22 wt or mutant Q64L, reduced the infection of DENV-1 HW and DENV-2 NGC about 60% in transfected cells in comparison to the expression of control GFP, and, by the contrary, the infection with DENV-2 16681 was not affected by the expression of these proteins (Fig. 6 A, C).

To further corroborate these results, we next evaluated the role on DENV infection of Rab11, other member of Rab GTPases involved in the last step of transport in the slow recycling route, from recycling endosomes to the plasma membrane [37]. Vero cells were transfected with the wt and DN mutant S25N versions of Rab11 fused to Green Lantern, a modified version of GFP, and then infected. The expression of the DN Rab11 S25N exerted a moderate inhibitory effect against DENV-1 HW and DENV-2 NGC, but the multiplication of DENV-2 16681 was not affected (Fig. 6B, C).

Finally, the presence of DENV-2 NGC and DENV-2 16681 particles in recycling and late endosomes, respectively, during the entry pathway into Vero cells was verified by colocalization of viral nucleocapsids with Rab11 and Rab7, markers of recycling and late endosomes, respectively. As seen in Fig. 6D, after 15 min of internalization DENV-2 16681 capsids were detected inside Rab7 positive vesicles whereas DENV-2 NGC capsids colocalized mainly with Rab11.

In conclusion, the three DENV strains appeared to locate first at the early endosomes, then DENV-2 16681 should be transported to late endosomes through the degradative pathway whereas DENV-1 HW and DENV-2 NGC employed the slow recycling route to the perinuclear recycling endosomes.

## Discussion

The investigation of the route of vesicular trafficking after virus uptake presented in this study showed a different involvement of cellular components not just between virus serotypes but also between virus strains of a same serotype. There are discrepancies in the results reported by different authors regarding the intracellular route followed by DENV particles after internalization until they arrive to the organelle where fusion takes place. After analysis of the requirement of Rab5 and Rab7 GTPase expression Krishnan et al. [5] concluded that the transport from early to late endosomes was not necesary for successful infection of HeLa cells with DENV-2 NGC. In apparent contradiction, the colocalization of DENV-2 16681 with lysosomes was reported in C6/36 cells, suggesting an endocytic pathway from early to late endosomes and lysosomes [8]. A similar conclusion about transport from Rab5-positive early endosomes to Rab7-decorated late endosomes for virus fusion was achieved by tracking fluorescently labeled DENV-2 PR159 S1 particles in living BSC-1 cells [13]. Interestingly, these authors reported that infectivity of DENV-2 PR159S1 was severely impaired in cells expressing DN Rab7 mutants, whereas the infectivity of DENV-2 NGC was unaffected, rising the suggestion that different virus strains may have distinct entry characteristics. Our results clearly support this hypothesis and offer an explanation to the discrepant results above mentioned when different DENV-2 strains were analyzed. A direct comparison of the involvement of functional Rab proteins for DENV multiplication in Vero cells showed that two DENV-2 strains, NGC and 16681, depend on functional Rab5 for successful infection, but when transport to late endosomes was blocked by the overexpression of a DN mutant of Rab7 the multiplication of DENV-2 16681 was severely reduced whereas DENV-2 NGC was not impaired (Fig. 4A–B). This result indicates that only DENV-2 16681 particles are transported to late endosomes or alternatively, that DENV-2 NGC is able to reach late endosomes in a Rab7-independent manner. Another experimental approach allowed us to support the first option, since when trafficking to late endosomes was inhibited by treatment with the drug wortmannin, only DENV-2 16681 particles were retained in Rab5 positive compartments (Fig. 5).

Noticeably, the infection of Vero cells with DENV-1 HW was also dependent of Rab5 functionality but independent of Rab7, suggesting that these virions do not reach late endosomes similarly as observed with DENV-2 NGC. Our previous studies demonstrated through molecular and biochemical approaches that different endocytic pathways were exploited by DENV-1 and DENV-2 for internalization into Vero cells, a phenomenon that is strain-independent as shown in Fig. 1. Then, the intracellular trafficking of DENV particles until membrane fusion appears to be variable and independent of the route for initial virion uptake.

Morphological and biochemical analysis of the endocytic pathway, as well as tracking studies of fluorescently labeled particles in live cells indicate that cargo delivered from the surface typically reaches early endosomes in less than 2 min after internalization, late endosomes in the perinuclear region after 10–12 min, and the lysosomes within 30–60 min [38]–[40]. Accordingly, viruses like lymphocytic choriomeningitis virus [18] and Uukuniemi virus [20] which penetrate from late endosomes, exhibit a half time of membrane penetration of 10–20 min while viruses fusing in early endosomes, such as vesicular stomatitis virus [25] or Semliki Forest virus [41], penetrate within 5 min after internalization. Through a susceptibility to ammonium chloride assay we showed that regardless the virus strain or serotype the first DENV particles reached the acid-dependent step 5 min after cell warming and half of the incoming infectious particles passed the ammonium chloride-sensitive step within 14–16 min post-internalization. These results are in line with previous fusion kinetics studies of other DENV-2 strains [6], [13], [42]. This late penetration kinetics indicates that in spite of their Rab7-independent multiplication, DENV-2 NGC and DENV-1 HW would be transported beyond the early endosomal compartments. By including in our study two members of the family of Rab GTPases that participate in regulation of transport through the recycling endosomes, we found that Rab22 reduced DENV-2 NGC and DENV-1 HW multiplication, while DENV-2 16681 was unaffected (Fig. 6A–C). This confirms that DENV-2 16681 may be transported to late endosomes and, in contrast, DENV-2 NGC would follow the recycling route. This differential incorporation of the two DENV-2 strains into the recycling or degradative pathways was further assessed by colocalization studies that demonstrated the presence of DENV-2 NGC viral capsids in Rab11-positive vesicles and DENV-2 16681 in Rab7-postitive vesicles at the half-time of virus penetration (Fig. 6D).

**Figure 7:**
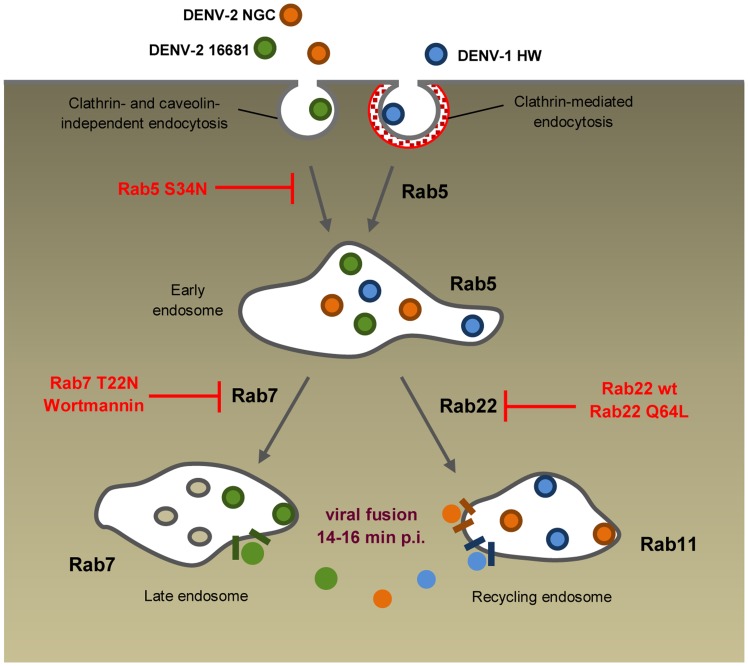
Model of infectious intracellular transport of DENV-1 HW, DENV-2 NGC and DENV-2 16681 in Vero cells. DENV-1 HW is internalized through the classical clathrin-mediated endocytic pathway, while DENV-2 NGC and DENV-2 16681 use a non-classical clathrin- and caveolin- independent route. These viruses are then transported in a Rab5-dependent manner to early endosomes. There, DENV-2 16681 is incorporated into late endosomes in a Rab7-dependent fashion and DENV-2 NGC and probably DENV-1 HW into recycling endosomes in a Rab22-dependent process. Viral fusion would take place in the corresponding organelles between 14–16 min p.i. releasing the viral nucleocapsids to the cytoplasm.

Early endosomes represent both the single entry point for internalized molecules and the first sorting station in the endocytic pathway. They are complex organelles with several tubular and vacuolar domains. The tubular domains, of 60–90 nm in diameter, contain most of the endosomal membrane and give rise to quick recycling vesicles as well as transport vesicles guiding to the slow recycling endosomes and to the trans-Golgi network. The vacuolar domains, 300–400 nm in diameter, contain most of the volume, the intralumenal vesicles, and large endocytosed particles [43]. Once delivered to early endosomes, most virus particles are too big to enter the tubular extensions and generally localized to the vacuolar domains being sorted to the degradative pathway [23]. Accordingly, only a few examples of viruses exploiting the recycling pathway to get a successful infection have been reported. So far, they include some members of *Polyomaviridae,* whose diameter is about 45 nm [44] and *Picornaviridae*, with 20–30 nm of diameter [45]. As DENV particles are in a size range between 40–50 nm of diameter [46], [47], there would not be any restriction for them to enter the narrow tubular extensions of early endosomes in route to the perinuclear recycling compartments. Interestingly, a recent report demonstrates that in addition to exposure to the acidic environment of the endosomes DENV particles require the presence of anionic lipids for fusion [42]. These authors propose that although viral particles are already exposed in early endosomes to acidic conditions enough to trigger conformational changes in the envelope protein, fusion cannot proceed until particles are delivered into an anionic lipid-enriched membrane. In this respect it is interesting to note that whereas late endosomes are enriched in the anionic lipid bis-(monoacylglycero)-phosphate [48], recycling endosomes are enriched in the anionic lipid phosphatidylserine [49], which was also reported to facilitate the fusion of different DENV serotypes and strains, including DENV-2 NGC [42].

Together, the results presented in this report demonstrate that in spite of the different internalization route among viral serotypes in Vero cells and regardless of the viral strain, DENV particles are first transported to early endosomes in a Rab5-dependent manner (Fig. 7). Then a Rab7-dependent pathway guides DENV-2 16681 to late endosomes, whereas a yet unknown sorting event controls the transport of DENV-2 NGC, and most probably DENV-1 HW, to the perinuclear recycling compartments where fusion membrane would take place releasing nucleocapsid into the cytoplasm. Further studies will establish which biochemical or physical forces are governing this differential sorting among DENV strains.

The conclusions presented in this work may also represent a new clue to understand viral pathogenesis. Toll-like receptors (TLR) capture signals derived from viral particles and subsequently initiate signalling for inflammatory cytokine response such as IL-8, IFN-α/β, TNF-α production [50]. It was shown that at the incipient stage of infection, after exposure of viral particles to the acidic endosomal pH, DENV RNA interacts with TLR3 molecules present in intracellular compartments and triggers IL-8 and IFN-α/β secretion [51]. On the other hand, a differential regulation of IFN induction by different strains of DENV was reported [52]. Future studies will determine whether this differential induction of the antiviral response is related to the distinct intracellular trafficking here reported among DENV strains, since a variation in the intracellular route may determine the encounter between viral RNA and TLRs early after virus uncoating.
